# Cryoballoon ablation of the left atrial posterior wall reduces recurrence of persistent atrial fibrillation in patients with non‐paroxysmal atrial fibrillation

**DOI:** 10.1002/joa3.12654

**Published:** 2021-11-05

**Authors:** Takatoshi Shigeta, Yasuteru Yamauchi, Yuichiro Sagawa, Atsuhito Oda, Shinichi Tachibana, Koji Sudo, Rena Nakamura, Kaoru Okishige, Masahiko Goya, Tetsuo Sasano

**Affiliations:** ^1^ Heart Center Japan Red Cross Yokohama City Bay Hospital Yokohama City Kanagawa Japan; ^2^ Department of Cardiovascular Medicine Tokyo Medical and Dental University Tokyo Japan

**Keywords:** ablation, atrial fibrillation, atrial tachycardia, cardiac surgical procedures, catheters

## Abstract

**Background:**

This study aimed to clarify the clinical outcomes of cryoballoon ablation of the left atrial (LA) posterior wall (LAPW), including the LA roof, in patients with non‐paroxysmal atrial fibrillation (AF).

**Methods:**

We analyzed the outcomes of 284 patients with non‐paroxysmal AF, of whom 210 underwent the cryoballoon ablation of the LAPW, including the LA roof, in addition to pulmonary vein isolation with a cryoballoon.

**Results:**

Complete conduction block at the LA roof was obtained in 95.7% (201/210) of patients, and LAPW was isolated in 83.3% (130/156) of patients. Over 372 (range, 208–477) days of follow‐up, atrial arrhythmia recurrence was observed in 84 (29.6%) patients, and atrial tachycardia (AT) recurrence accounted for 27.4% of cases. The prevalence of LA roof cryoballoon ablation was significantly higher in patients without recurrence than in those with recurrence (78.6% vs. 63.1%, respectively; *p* = .01), especially those with persistent AF recurrence (77.0% vs. 55.0%, *p* = .01). No significant difference was found in the prevalence of AT recurrence between patients who had undergone additional LAPW ablation and those who had not. Durable LA roof lesions were confirmed in 29 (72.5%) of 40 patients who underwent redo ablation.

**Conclusions:**

Cryoballoon ablation of the LAPW leads to a sufficient acute success rate of complete conduction block and durable lesions of the LA roof without increasing AT recurrence risk. The prevalence of persistent AF recurrence decreases after additional cryoballoon ablation of the LAPW in patients with non‐paroxysmal AF.

## Graphical abstract

Cryoballoon ablation of the left atrial roof or the left atrial posterior wall could be performed safely. It brings sufficient efficacy in terms of reducing the recurrence as a persistent form of atrial fibrillation.

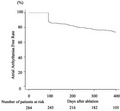

## 1 INTRODUCTION

The recurrence of atrial fibrillation (AF) can be controlled by the established treatment of pulmonary vein isolation (PVI)^1^; however, the effect of PVI appears to be insufficient in patients with non‐paroxysmal AF. Additional strategies for AF catheter ablation have been considered in previous investigations, and linear ablation of the left atrial (LA) roof line and mitral isthmus (MI) line is one of the additional therapeutic methods of catheter ablation.^2^ Atrial tachycardia (AT) recurrence has been reported as an undesirable outcome after catheter ablation for AF, which might be attributable to linear ablations.^3^ Ablation of the LA roof with a cryoballoon in addition to PVI was investigated as a novel method for LA roof line ablation.^4^ However, detailed clinical outcomes, including the type of recurrence, have not been fully examined. Thus, this study aimed to clarify the details after catheter ablation in patients with non‐paroxysmal AF, especially those who underwent the cryoballoon ablation of the LA posterior wall (LAPW), including the LA roof.

## 2 METHODS

### 2.1 Study patients

This single‐center retrospective study included consecutive patients with non‐paroxysmal AF who underwent cryoballoon ablation with PVI between September 2014 and June 2019. Patients who underwent heart surgery were excluded because the surgery could have contributed to the AT occurrence. Patients who underwent LA roof line or LAPW bottom line ablation using a radiofrequency (RF) catheter were also excluded because the effect of the ablation was considered to vary between LA roof line or LAPW bottom line ablation performed with a cryoballoon and that performed with an RF catheter. Patients who did not undergo clinical follow‐up of more than 3 months were excluded from the analysis. We hypothesized that LAPW ablation, including cryoballoon ablation of the LA roof in addition to PVI, is effective in reducing the recurrence of persistent AF in patients with non‐paroxysmal AF without inducing AT. To compare the effect of additional LAPW ablation with that of only applying PVI, only patients who did not undergo MI ablation were analyzed. All patients underwent three‐dimensional computed tomography and transthoracic echocardiography before ablation. Paroxysmal AF was defined as AF terminating within 7 days according to the previously reported definition^5^; hence, non‐paroxysmal AF was defined as AF persisting for more than 7 days. Baseline demographic characteristics, comorbidities, and medications were recorded.

### 2.2 Index ablation procedure

The ablation procedure was performed as previously described.^6^ Briefly, the procedure was performed under general anesthesia. A single transseptal puncture was made under fluoroscopic and intracardiac echocardiographic (Acuson and AcuNav; Biosense Webster) guidance. A 28‐mm cryoballoon catheter (Arctic Front Advance; Medtronic) was introduced into the LA through a steerable sheath (Flexcath Advance; Medtronic) with a circular mapping catheter (Achieve; Medtronic). Cryothermal energy was applied through a cryoballoon occluding each pulmonary vein (PV). The compound motor action potentials of the diaphragm provoked by phrenic nerve pacing were continuously monitored during ablation of the right PVs. When a successful PVI was not achieved solely by a cryoballoon, a touch‐up ablation with an RF catheter (FlexAbility or TactiCath; Abbott) was performed.

**FIGURE 1:**
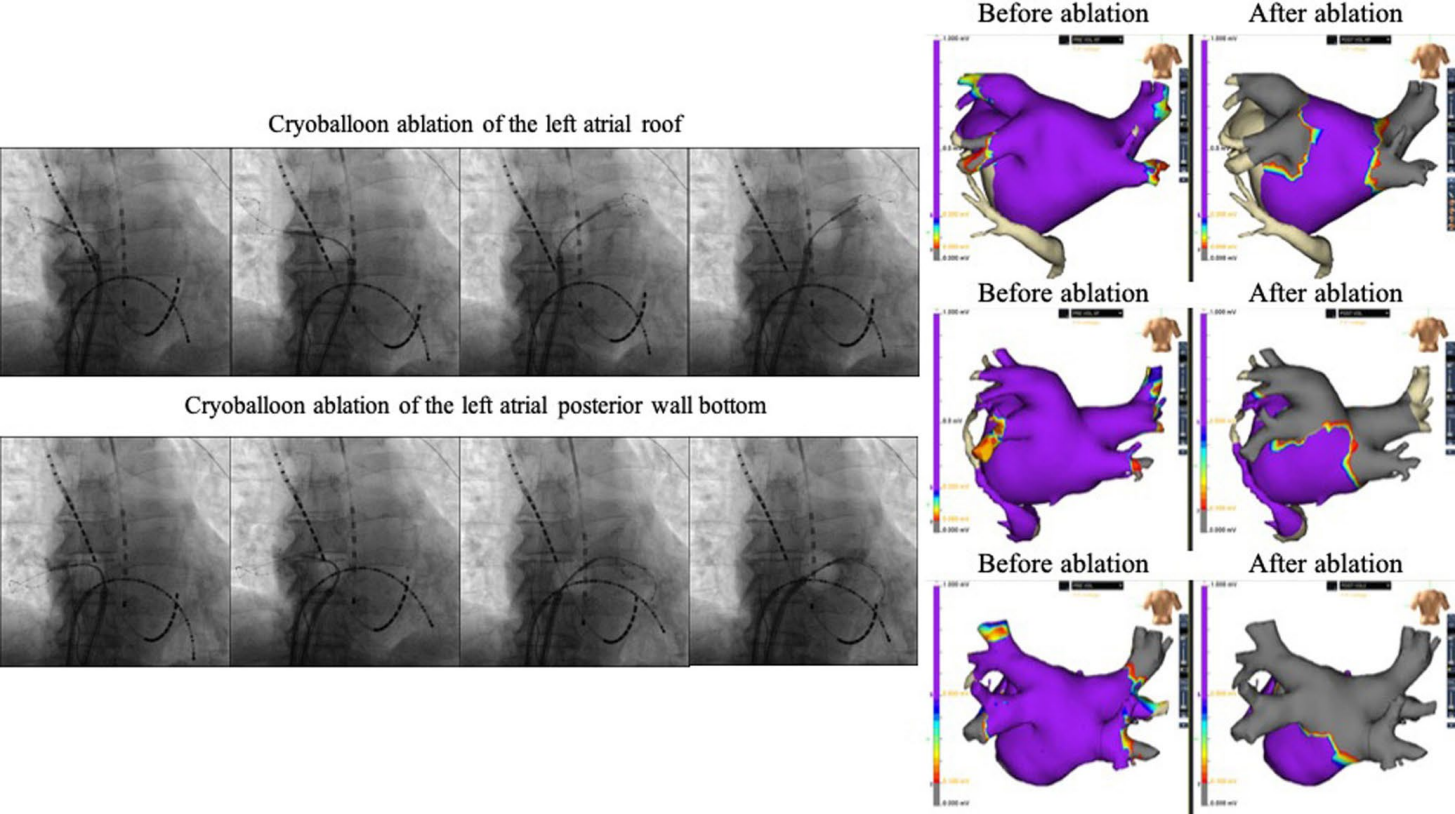
(Left panel) Fluoroscopic images during cryoballoon ablation of the (top) LA roof and (bottom) LAPW bottom (PA view). The cryoballoon is shifted gradually on the LA roof or the LAPW bottom from the left superior pulmonary vein to the right superior pulmonary vein or from the left inferior pulmonary vein to the right inferior pulmonary vein in an overlapping manner. (Right panel) Voltage map before and after the ablation procedure of a representative case in each therapeutic strategy. Upper panels show the case where we performed only PVI with a cryoballoon. The normal voltage area was left on the LAPW after the ablation. Middle panels show the case where we performed cryoballoon ablation of the LA roof without bottom line ablation. The normal voltage area left on the LAPW became smaller after the ablation. Lower panels show the case where we performed cryoballoon ablation of the LAPW in addition to PVI. The normal voltage area on the LAPW was confirmed before the ablation area became the scar area after the ablation, which indicates LAPW isolation. LA, left atrial; LAPW, left atrial posterior wall; PVI, pulmonary vein isolation

Additional ablations were performed according to the operator's decision. When LA roof cryoballoon ablation was performed, an Achieve catheter was inserted into the left and right superior PVs, and a cryoballoon was shifted along the LA roof by changing the direction of the steerable sheath and adjusting the position of the cryoballoon and sheath so that each cryoballoon location overlapped with the previous cryoballoon location. Cryoballoon ablation of the bottom line of the LAPW was performed in the same manner, with the Achieve catheter positioned in each inferior PV (Figure 1). Cryothermal energy was applied for 180–240 s according to the operator's decision. Touch‐up ablation of the LAPW with an RF catheter was permitted. Cryoballoon ablation was performed under luminal esophageal temperature (LET) monitoring using esophageal temperature probes (Esophaster; Japan Lifeline) with a cutoff value of 15°C. RF applications were also prematurely interrupted when the LET reached 39°C. Linear ablation at the cavotricuspid isthmus (CTI) was performed by delivering RF energy from the tricuspid annulus to the inferior vena cava in a point‐by‐point fashion. After each linear ablation, the status of the conduction block was confirmed by an electrophysiological method, an activation map, and a voltage map created with an electrical impedance‐based mapping system (Ensite NavX; Abbott). PVI was confirmed using a duodecapolar circular mapping catheter (EPstar Libero; Japan Lifeline). The voltage map before and after the ablation procedure of the representative cases is depicted in Figure 1. All procedures were performed by either of the two main operators skilled with the technique

### 2.3 Clinical follow‐up

Anti‐arrhythmic drugs (AADs) were prescribed after ablation at the discretion of the patient's attending cardiologist. Twelve‐lead electrocardiograms (ECGs) were recorded at every follow‐up visit or an emergency visit owing to symptoms suggestive of an arrhythmia recurrence. In addition, 24‐hour Holter ambulatory ECG monitoring was performed to detect the recurrence of paroxysmal arrhythmias. An arrhythmia recurrence was defined as any documented atrial arrhythmia lasting longer than 30 s after the initial 90‐day blanking period.

### 2.4 Redo ablation procedure

The repeat ablation procedure was performed in a manner similar to the index one. When AT persisted at the beginning of the procedure, it was mapped using an EPstar Libero and Ensite NavX system. Along with electrophysiological findings, catheters and systems were used to confirm the status of the lesions created in the index ablation procedure. Based on the operator's judgment, ablation was performed for novel or recurring lesions.

### 2.5 Statistical analysis

Continuous variables are expressed as mean ± standard deviation, or median with the interquartile range. Categorical variables are reported as numbers and percentages. Comparisons were made using Fisher's test, Student's *t*‐test, Mann–Whitney *U* test, one‐way analysis of variance test, Kruskal–Wallis test, or log‐rank test. Holm method, Dunnett's test, or Steel's multiple comparison test was performed for the post hoc test. Cox regression analysis was also used to evaluate each recurrence type after catheter ablation in the multivariate models. A *p*‐value of .10 was required for entry into the model, and a stepwise method was utilized to detect any predictors. A *p*‐value <.05 was considered statistically significant. All analyses were performed using the EZR software (Saitama Medical Center, Jichi Medical University), a graphical user interface of R (The R Foundation for Statistical Computing).

## 3 RESULTS

### 3.1 Study population

Forty‐three patients were excluded because of previous heart surgery (nine patients), LA roof line or LAPW bottom line ablation performed with an RF catheter (nine patients), and insufficient clinical follow‐up after catheter ablation (25 patients). Of the remaining 429 patients, 145 underwent MI ablation. Finally, data of 284 patients with non‐paroxysmal AF were analyzed. The baseline patient characteristics are shown in Table 1 and compared between patients who underwent only PVI with a cryoballoon, those who underwent cryoballoon ablation of the LA roof in addition to PVI, and those who underwent cryoballoon ablation of the LA roof and the LAPW bottom in addition to PVI (Table 2). Four patients had right middle PVs, and 19 patients had left common PVs.

**TABLE 1:** Patient characteristics and index ablation results

	Study cohort	Recurrence			AT recurrence			AF recurrence			Persistent AF recurrence		
	(*n* = 284)	– (*n* = 200)	+ (*n* = 84)	*p*	– (*n* = 261)	+ (*n* = 23)	*p*	– (*n* = 216)	+ (*n* = 68)	*p*	– (*n* = 244)	+ (*n* = 40)	*p*
Age (years)	65.3 ± 10.6	64.6 ± 10.3	66.9 ± 11.0	.11	64.9 ± 10.6	70.3 ± 8.7	.02	65.1 ± 10.4	66.0 ± 11.2	.56	65.0 ± 10.5	67.3 ± 10.6	.21
Male sex	223 (78.5)	160 (79.6)	64 (76.2)	.53	207 (79.0)	17 (73.9)	.60	172 (79.6)	51 (75.0)	.40	192 (78.7)	31 (77.5)	.84
Body mass index (kg/m^2^)	25.1 ± 4.2	25.2 ± 4.3	24.8 ± 3.9	.49	25.0 ± 4.2	25.3 ± 4.2	.78	25.1 ± 4.3	24.8 ± 3.8	.51	25.2 ± 4.3	24.1 ± 3.3	.11
AF duration (months)	4.0 [2.0–12.5]	3.0 [2.0–10.0]	6.0 [2.0–29.0]	.03	4.0 [2.0–12.8]	2.0 [1.0–11.0]	.12	3.0 [2.0–10.0]	8.0 [2.0–32.0]	<.01	3.0 [2.0–12.0]	5.5 [2.0–32.3]	.07
Long‐standing persistent AF	77 (27.1)	44 (22.1)	33 (40.2)	<.01	71 (27.5)	6 (26.1)	1.00	48 (22.4)	29 (43.9)	<.01	62 (25.7)	15 (38.5)	.12
Hypertension	131 (46.1)	94 (46.8)	38 (45.2)	.90	123 (46.9)	9 (39.1)	.52	98 (45.4)	33 (48.5)	.68	111 (45.5)	20 (50.0)	.61
Diabetes	47 (16.6)	30 (14.9)	17 (20.2)	.30	43 (16.4)	4 (17.4)	1.00	32 (14.8)	15 (22.1)	.19	39 (16.0)	8 (20.0)	.50
Chronic heart failure	34 (12.0)	20 (10.0)	15 (17.9)	.07	28 (10.7)	7 (30.4)	.01	24 (11.1)	10 (14.7)	.40	27 (11.1)	7 (17.5)	.29
Prior stroke/TIA	22 (7.8)	16 (8.0)	6 (7.1)	1.00	20 (7.6)	2 (8.7)	.70	18 (8.3)	4 (5.9)	.61	18 (7.4)	4 (10.0)	.53
LVEF (%)	61.2 ± 11.0	61.0 ± 10.6	61.8 ± 12.0	.59	61.8 ± 10.2	55.1 ± 16.8	<.01	60.6 ± 11.2	63.3 ± 10.1	.09	61.1 ± 11.0	62.1 ± 10.7	.61
LA diameter (mm)	44.2 ± 6.4	43.9 ± 6.3	45.1 ± 6.7	.15	44.0 ± 6.2	46.3 ± 8.4	.10	44.1 ± 6.5	44.5 ± 6.1	.66	44.2 ± 6.5	44.3 ± 5.9	.92
LCPV or RMPV	23 (8.1)	15 (7.5)	8 (9.5)	.64	20 (7.6)	3 (13.0)	.41	17 (7.9)	6 (8.8)	.80	19 (7.8)	4 (10.0)	.55
Treatment with AADs	72 (25.4)	32 (15.9)	40 (47.6)	<.01	61 (23.3)	11 (47.8)	.02	39 (18.1)	33 (48.5)	<.01	51 (20.9)	21 (52.5)	<.01
Roof line	210 (73.9)	158 (78.6)	53 (63.1)	.01	193 (73.7)	18 (78.3)	.81	169 (78.2)	41 (60.3)	<.01	188 (77.0)	22 (55.0)	.01
Bottom line	156 (54.9)	118 (58.7)	39 (46.4)	.07	143 (54.6)	10 (71.4)	.66	127 (58.8)	29 (42.6)	.03	142 (58.2)	14 (35.0)	.01
Touch‐up ablation for LAPW isolation	42 (14.8)	31 (15.9)	12 (16.0)	1.00	37 (14.9)	14 (60.9)	.13	34 (16.3)	8 (13.3)	.69	39 (16.8)	3 (8.1)	.23
CTI ablation	276 (97.2)	197 (98.0)	80 (95.2)	.24	255 (97.3)	22 (95.7)	.50	211 (97.7)	65 (95.6)	.40	238 (97.5)	38 (95.0)	.31

Note

Values are presented as mean ± standard deviation, *n* (%), or median [interquartile range].

Abbreviations: AADs, anti‐arrhythmic drugs; AF, atrial fibrillation; AT, atrial tachycardia; CTI, cavotricuspid isthmus; LA, left atrial; LAPW, left atrial posterior wall; LCPV, left common pulmonary vein; LVEF, left ventricular ejection fraction; MI, mitral isthmus; RMPV, right middle pulmonary vein; TIA, transient ischemic attack.

**TABLE 2:** Comparison of patient characteristics and index ablation results of patients with and without cryoballoon ablation of the LA roof

	Patients with PVI only	Patients with PVI + roof line ablation	Patients with PVI + LAPW ablation	*p*
	(*n* = 74)	(*n* = 54)	(*n* = 156)	
Age (years)	67.8 ± 11.2	65.1 ± 10.7	64.2 ± 10.0	.048*
Male sex	54 (73.0)	44 (81.5)	125 (80.1)	.41
Body mass index (kg/m^2^)	25.0 ± 4.5	24.8 ± 3.4	25.1 ± 4.3	.88
AF duration (months)	3.0 [2.0–11.3]	4.0 [2.0–10.0]	4.0 [2.0–24.0]	.44
Hypertension	31 (41.9)	27 (50.0)	73 (46.8)	.65
Diabetes	11 (14.9)	13 (24.1)	23 (14.7)	.27
Chronic heart failure	11 (14.9)	4 (7.4)	19 (12.2)	.49
Prior stroke/TIA	7 (9.5)	2 (3.7)	13 (8.3)	.43
LVEF (%)	61.6 ± 10.4	63.9 ± 9.0	60.1 ± 11.7	.09
LA diameter (mm)	44.7 ± 6.5	44.1 ± 5.9	44.0 ± 6.6	.77
LCPV or RMPV	4 (5.4)	2 (3.8)	17 (10.9)	.21
Treatment with AADs	23 (31.1)	10 (18.5)	39 (25.0)	.28
Total ablation time (min)	18.9 ± 6.8	34.9 ± 10.0	46.1 ± 11.6	<.01* ^,^ ^+^ ^,^ ^§^
Total procedure time (min)	115.4 ± 30.9	148.9 ± 40.7	161.4 ± 34.5	<.01* ^,^ ^+^ ^,^ ^§^
Touch‐up ablation for LAPW isolation	0 (0.0)	4 (7.5)	38 (24.4)	<.01* ^,^ ^+^ ^,^ ^§^
CTI ablation	66 (89.2)	54 (100.0)	156 (100.0)	<.01* ^,^ ^+^

Note

Values are presented as mean ± standard deviation, *n* (%), or median [interquartile range].

Abbreviations: AADs, anti‐arrhythmic drugs; AF, atrial fibrillation; CTI, cavotricuspid isthmus; LA, left atrial; LAPW, left atrial posterior wall; LCPV, left common pulmonary vein; LVEF, left ventricular ejection fraction; RMPV, right middle pulmonary vein; TIA, transient ischemic attack; PVI, pulmonary vein isolation.

* *p* < .05 between patients with PVI only and those with PVI + LAPW ablation.

+ *p* < .05 between patients with PVI only and those with PVI + roof line ablation.

§ *p* < .05 between patients with PVI + roof line ablation and those with PVI + LAPW ablation.

### 3.2 Index ablation results

The procedural characteristics during the ablations are shown in Table 1. All PVs were successfully isolated in all patients, and touch‐up ablation with RF energy was required in 61 (21.5%) of 284 patients (left superior PV, 4 patients; left inferior PV, 17 patients; right superior PV, 12 patients; right inferior PV, 39 patients; left common PV, 2 patients). Complete conduction block at the LA roof was obtained in 201 (95.7%) of 210 patients. The prevalence of complete conduction block at the LAPW bottom line, namely, LAPW isolation, including the case with touch‐up ablation, was 83.3% (130/156 patients). Touch‐up ablation with RF energy was performed in 10 patients (4.8%) for the LA roof line and 37 patients (23.7%) for the LAPW bottom line. The left atrial diameter (LAD) was significantly smaller in those with complete conduction block obtained by only cryoballoon ablation than in those without (LA roof line: 43.6 ± 6.0 mm for those with complete conduction block vs. 50.7 ± 8.7 mm for those without complete conduction block, *p* < .01; LAPW bottom line: 43.3 ± 6.2 mm for those with complete conduction block vs. 46.9 ± 7.2 mm for those without complete conduction block, *p* < .01). CTI ablation was performed in 276 patients, of whom 264 (95.7%) had complete conduction block. Furthermore, AF termination could be confirmed in 14 of 176 patients in whom we performed ablation during AF (7 in PVI, 6 in LA roof line, and 1 in LAPW bottom line).

Transient and persistent phrenic nerve injuries were observed in 27 (9.5%) and 11 (3.9%) patients, respectively. Phrenic nerve injury eventually recovered in all cases. Two (0.7%) patients had cardiac tamponade that required pericardiocentesis. One patient developed arteriovenous fistula requiring surgical repair. No other procedural complications, such as stroke or symptomatic esophageal complications, were observed (Table 3).

**TABLE 3:** Procedural complications during the index ablation procedure

Arteriovenous fistula	1 (0.4)
Cardiac tamponade	2 (0.7)
Transient phrenic nerve injury	27 (9.5)
Persistent phrenic nerve injury	11 (3.9)
Stroke	0 (0.0)
Symptomatic esophageal complications	0 (0.0)

### 3.3 Clinical outcomes

**FIGURE 2:**
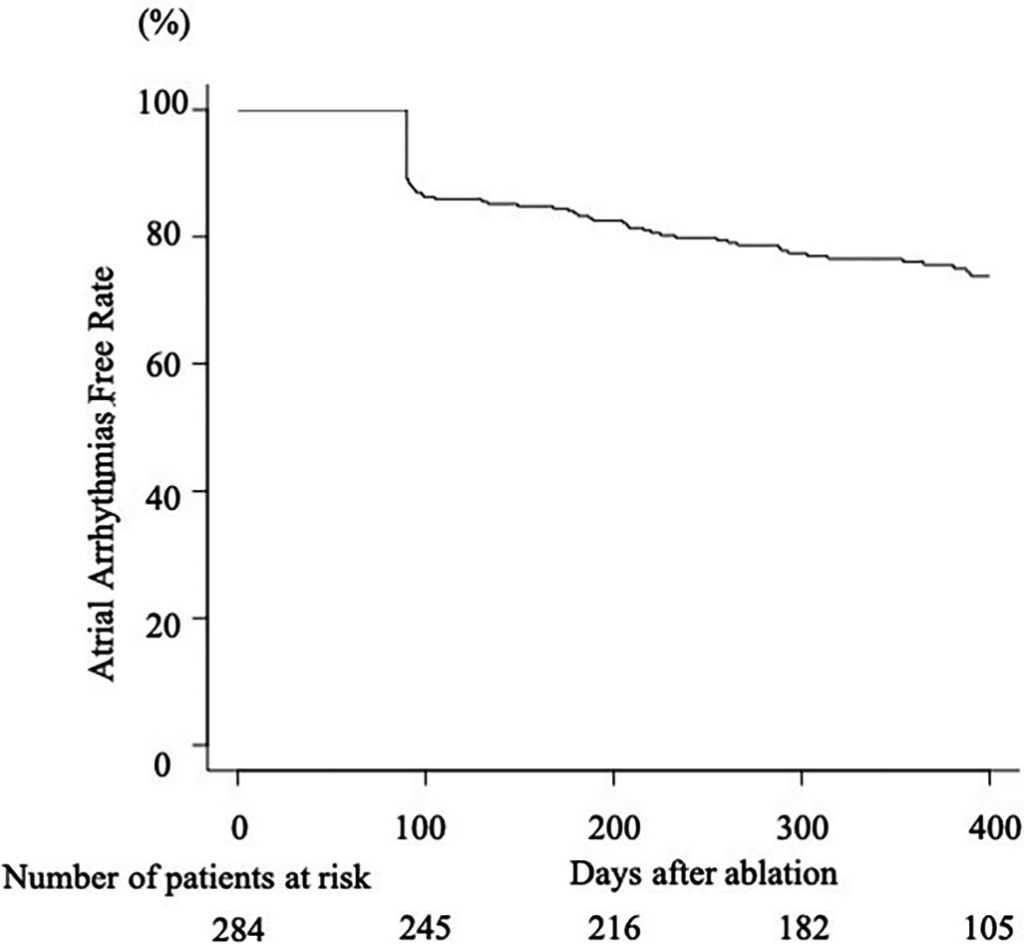
Kaplan–Meier curve of atrial arrhythmia recurrence after index ablation

**FIGURE 3:**
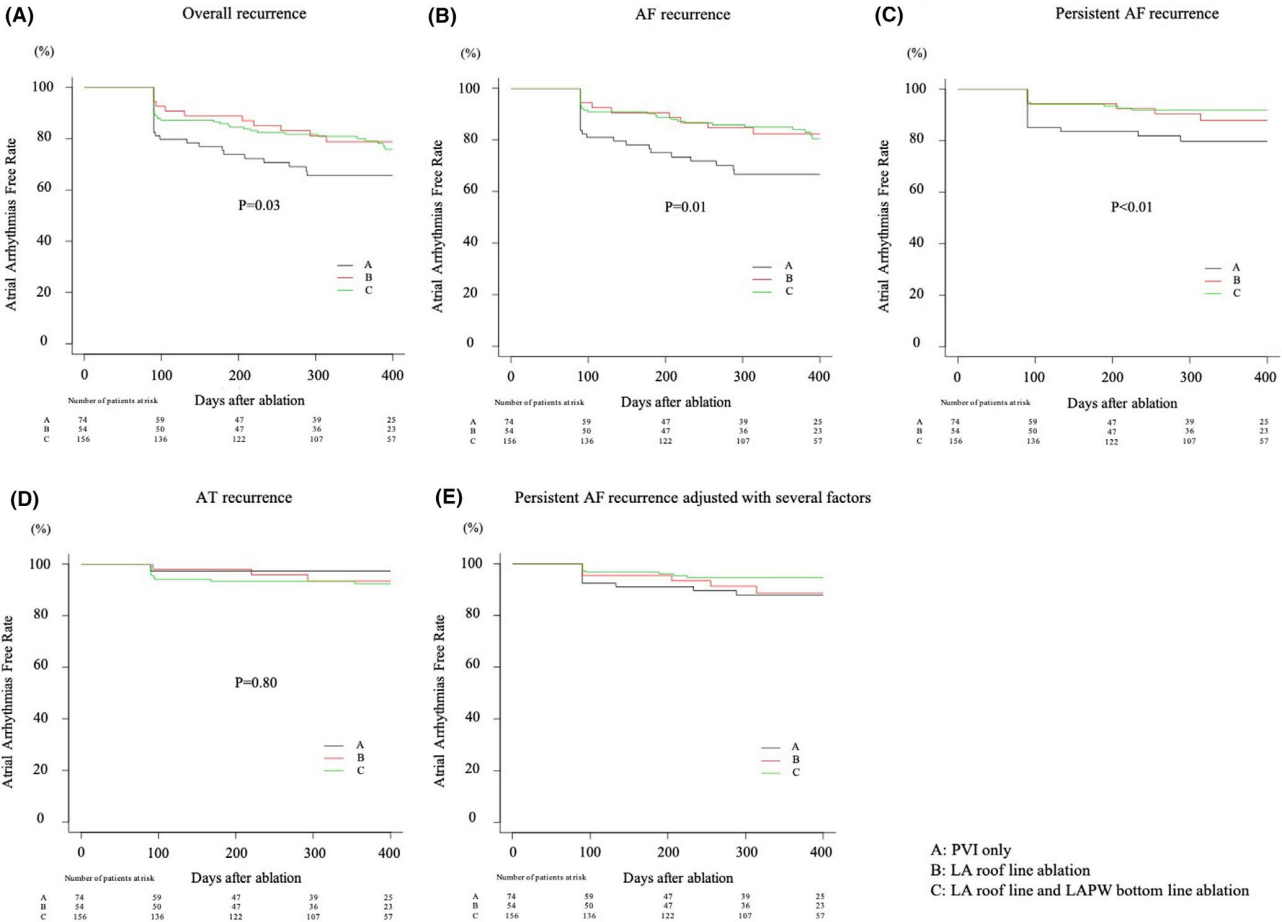
Kaplan–Meier curves of (A) overall atrial arrhythmia recurrence, (B) AF recurrence, (C) persistent AF recurrence, (D) AT recurrence, and (E) persistent AF recurrence adjusted with several factors (AAD use, age, sex, body mass index, LAD, and AF duration) after the index ablation. The clinical outcomes for each recurrence pattern were compared among the three groups (Group A, patients who underwent only PVI; Group B, patients who underwent LA roof line ablation in addition to PVI; Group C, patients who underwent LA roof line and LAPW bottom line ablation in addition to PVI). The 12‐month Kaplan–Meier event‐free rate estimates were as follows: 65.7% versus 78.7% versus 79.1% (overall recurrence), 66.7% versus 82.4% versus 84.0% (AF recurrence), 79.8% versus 87.8% versus 91.9% (persistent AF recurrence), and 97.3% versus 93.4% versus 92.4% (AT recurrence). According to the Holm method, significant differences were found between patients who had undergone PVI only and those who had undergone LA roof line and LAPW bottom line ablation in terms of overall recurrence (*p* = .04), AF recurrence (*p* = .01), and persistent AF recurrence (*p* = .01). AADs, anti‐arrhythmic drugs; AT, atrial tachycardia; AF, atrial fibrillation; LA, left atrial; LAD, left atrial diameter; LAPW, left atrial posterior wall; PVI, pulmonary vein isolation

The median follow‐up duration was 372 [interquartile range, 208–477] days. Atrial arrhythmia recurrence was observed in 84 (29.6%) of 284 patients, and the 12‐month Kaplan–Meier event‐free rate was 75.6% (Figure 2). Of 284 patients, AT and AF recurrence were confirmed in 23 (8.1%) and 68 (23.9%) patients, respectively. The persistent type accounted for 58.8% of AF recurrences (40/68 patients). Table 1 shows the comparisons between patients based on recurrence, with each recurrence pattern in addition to overall recurrence. The prevalence of LA roof line ablation and additional LAPW bottom line ablation was higher in patients without recurrence than in those with recurrence, and when we divided the study patients into three groups (Group A, patients who underwent only PVI; Group B, patients who underwent LA roof line ablation in addition to PVI; Group C, patients who underwent LA roof line and LAPW bottom line ablation in addition to PVI), the 12‐month Kaplan–Meier event‐free rate was 65.7% in Group A, 78.7% in Group B, and 79.1% in Group C (Figure 3). When the recurrence rate was analyzed according to recurrence patterns, AF recurrence rate, particularly the persistent AF recurrence rate, was significantly lower in patients who had undergone cryoballoon ablation of the LAPW than in those who had undergone only PVI. The 12‐month Kaplan–Meier event‐free rates were 66.7% (AF recurrence) and 79.8% (persistent AF recurrence) in Group A, 82.4% (AF recurrence) and 87.8% (persistent AF recurrence) in Group B, and 84.0% (AF recurrence) and 91.9% (persistent AF recurrence) in Group C (Figure 3). According to the multivariate analysis using Cox regression analysis, additional LAPW bottom line ablation was identified as a predictor for freedom of persistent AF recurrence in addition to AAD use and AF duration (Table 3). Although the prevalence of AF recurrence was significantly lower in patients who had undergone additional LAPW bottom line ablation than in those who had not, additional LAPW bottom line ablation was not identified as a reliable predictor of AF recurrence after Cox regression analysis. Concerning AT recurrence, multivariate analysis with Cox regression analysis revealed only AAD use as a predictor of AT recurrence (Table 4). LAPW ablation, including cryoballoon ablation of the LA roof, was not associated with AT recurrence. Even when we performed further analysis among patients who had not undergone touch‐up ablation for either PVI or LAPW ablation, the 12‐month Kaplan–Meier event‐free rate regarding persistent AF recurrence was significantly higher in patients who had undergone cryoballoon ablation of the LAPW than in those who had not (93.0% for those with additional LAPW ablation vs. 80.8% for those with only PVI, *p* = .03). However, AT recurrence between those two groups was not significantly different (96.4% for those with additional LAPW ablation vs. 96.1% for those with only PVI, *p* = .80). Atrial arrhythmia recurrence tended to be higher in the patients in whom AF termination could be observed during ablation (the 12‐month Kaplan–Meier event‐free rates were 85.1% for the patients in whom AF termination could be observed vs. 69.7% for those in whom AF termination could not be observed, *p* = .09). The A wave velocity of the trans‐mitral flow was measured by transthoracic echocardiography in 147 of 284 patients, and a significant difference was found between patients who underwent cryoballoon ablation of the LAPW and those who did not (0.52 ± 0.13 m/s for patients who underwent cryoballoon ablation of the LAPW vs. 0.65 ± 0.25 m/s for those who did not, *p* < .01).

**TABLE 4:** Univariate and multivariate analyses results for persistent AF recurrence and AT recurrence

	Univariate			Multivariate		
	HR	95% CI	*p*	HR	95% CI	*p*
Predictors of persistent AF recurrence						
Long‐standing persistent AF	1.94	1.01–3.73	.045			
AF duration	1.01	1.01–1.02	<.01	1.01	1.01–1.02	<.01
Treatment with anti‐arrhythmic drugs	4.09	2.19–7.65	<.01	4.04	2.11–7.70	<.01
Cryoballoon ablation of the LA roof	0.41	0.22–0.77	.01			
Cryoballoon ablation of the LAPW bottom	0.45	0.23–0.86	.02	0.41	0.21–0.81	.01
Predictors of AT recurrence						
Age	1.04	1.00–1.09	.07			
Chronic heart failure	2.75	1.12–6.74	.03			
Treatment with anti‐arrhythmic drugs	3.88	1.70–8.90	<.01	3.86	1.68–8.85	<.01
Left atrial diameter	1.06	1.00–1.13	.06			

Abbreviations: AF, atrial fibrillation; AT, atrial tachycardia; CI, confidence interval; HR, hazards ratio; LA, left atrial; LAPW, left atrial posterior wall.

### 3.4 Redo ablation results

Among the 84 patients exhibiting recurrence after the index catheter ablation procedure, 55 patients underwent a redo ablation procedure. Among the 18 patients with AT recurrence who underwent a redo ablation, AT persisted at the beginning of the procedure in 12 (66.7%) patients, and the type of AT was identified in all patients. Perimitral AT circulating around the mitral annulus was the most frequently confirmed type (five patients). Among these five patients with perimitral AT recurrence, cryoballoon ablation of the LA roof was performed in the index ablation procedure of four patients. Roof‐dependent AT, which contained the LA roof in the reentrant circuit, was observed in three patients; all of them had undergone cryoballoon ablation of the LA roof in the index ablation procedure. PV gap‐related AT was confirmed in two patients, and CTI‐dependent AT was confirmed in the other two patients.

**FIGURE 4:**
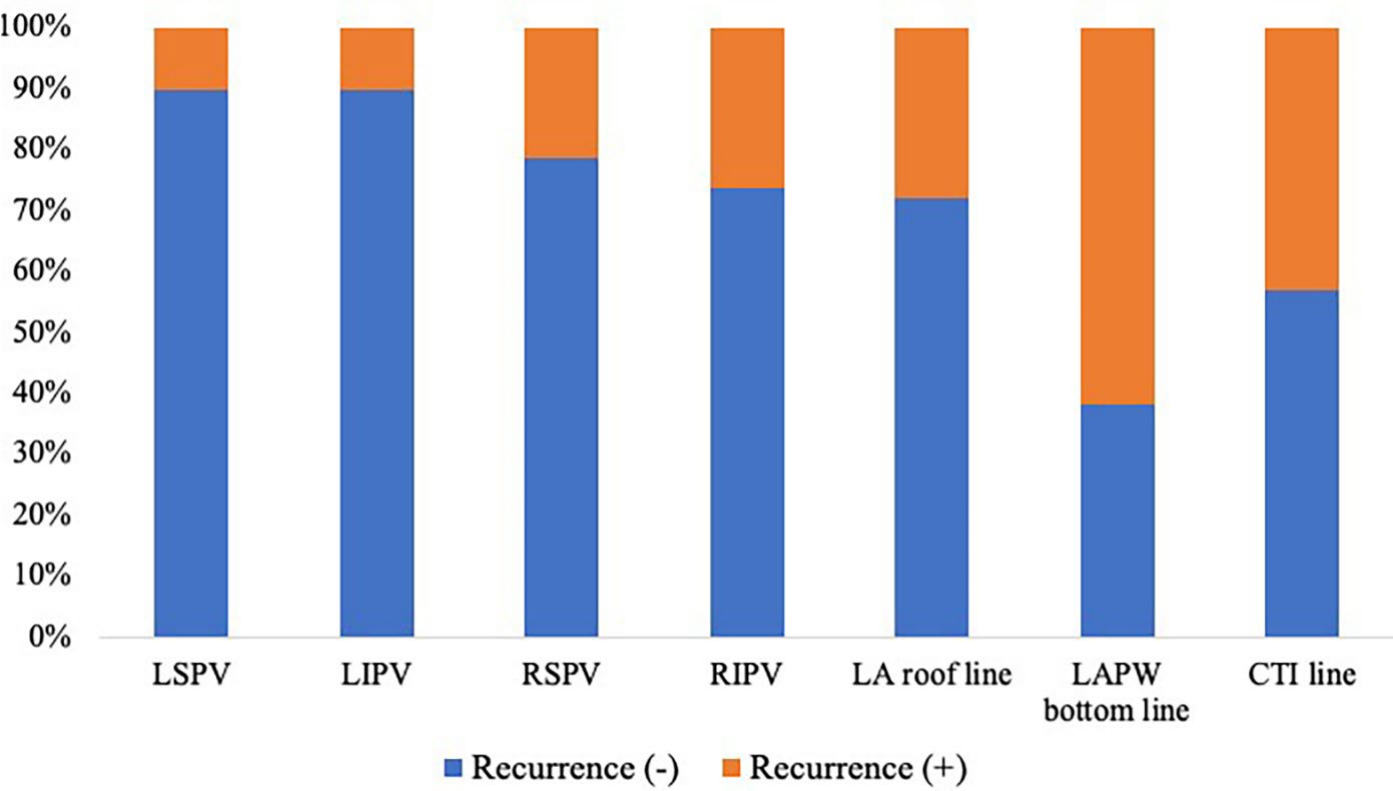
Percentage of durable lesions confirmed at the beginning of the redo ablation procedure. The analysis of PV durability was performed in 55 patients, including three patients with an LCPV who underwent redo ablation procedures. PV recurrence was confirmed in 6, 7, 15, and 18 patients at the LSPV, LIPV, RSPV, and RIPV, respectively. The durability of the LA roof line, LAPW bottom line, and CTI line was analyzed in patients who had obtained complete conduction block in the index ablation procedure (40 patients for the LA roof line, 29 patients for the LAPW bottom line, and 52 patients for the CTI line). CTI, cavotricuspid isthmus; LA, left atrial; LAPW, left atrial posterior wall; LCPV, left common pulmonary vein; LIPV, left inferior pulmonary vein; LSPV, left superior pulmonary vein; PV, pulmonary vein; RIPV, right inferior pulmonary vein; RSPV, right superior pulmonary vein

The durability of each lesion created in the index ablation procedure is described in Figure 4. The prevalence of patients in whom all PVs were confirmed to be durable was 41.8% (23/55 patients). PV reconnections were observed in 46 of 217 PVs. When we compared the initial ablation results between reconnected PVs and those without reconnections, the nadir balloon temperature during freezing was significantly higher in reconnected PVs (−45.2 ± 6.8℃ for reconnected PVs vs. −51.0±7.2℃ for those without reconnections, *p* < .01), and time to isolation was significantly longer in reconnected PVs (59.3 ± 33.8℃ for reconnected PVs vs. 41.8 ± 26.0℃ for those without reconnections, *p* = .01). Complete conduction blocks at the LA roof, LAPW bottom, and CTI were confirmed to be durable in 72.5% (29/40), 48.3% (14/29), and 61.5% (32/52) of patients, respectively. The rates of lesion durability of the PVI and LA roof lines without touch‐up ablations were 66.7% (28/42) and 78.3% (18/23) of patients, respectively. When the percentages of lesion durability between patients with AT recurrence (30.8%) and those without AT recurrence (52.2%) were compared, the prevalence of LA roof line recurrence was comparable (*p* = .30). At the beginning of the session, the ratio of the scar area to the LAPW area was significantly higher (*p* < .01) in patients who had undergone cryoballoon ablation of the LAPW in the index ablation (64.7 [44.0–82.7] %) than in those who had not (12.2 [1.8–26.4] %).

Additional linear ablation at the LAPW, including the LA roof line, was performed in 45 (93.8%) of 48 patients whose LAPW was not isolated at the beginning of the redo ablation procedure. MI ablation was performed in 23 (41.8%) of 55 patients. Of the 53 patients who underwent follow‐up after the redo ablation procedure, 11 patients underwent further ablation procedure, and 43 (81.1%) were finally free from atrial arrhythmias. The types of final recurrence in the residual 10 patients were paroxysmal AF in four patients, paroxysmal AT in one patient, and persistent AF in five patients.

## 4 DISCUSSION

This study investigated the details after catheter ablation in patients with non‐paroxysmal AF, particularly those who underwent LAPW cryoballoon ablation, including the LA roof. The main results showed that cryoballoon ablation of the LAPW was not associated with a high incidence of ATs during follow‐up. An LA roof‐dependent AT was observed in only 1.4% of the patients. Moreover, clinical outcomes were improved after the ablation procedure was compared with the strategy without LAPW ablation, especially regarding the recurrence of the persistent type of AF. Details of recurrence patterns, including the type of AT, and the durability of lesions created in the index ablation procedure were also investigated in this single‐center large cohort study.

PVI has been established as the gold standard for treating AF, and cryoballoon ablation has emerged as a novel ablation method instead of the conventional method utilizing RF energy^7^; however, its efficacy may be limited, especially in patients with non‐paroxysmal AF. Although their impact on clinical outcomes has not been sufficiently proven, various therapeutic approaches have been investigated for AF catheter ablation, such as linear ablation of the LA roof line and MI line. In particular, linear ablation that targets the LAPW, including isolation of the LA roof line and LAPW bottom line, has been a prominent focus.^2^ Recent studies have demonstrated that a cryoballoon could be utilized for linear ablation at the LAPW, and this additional ablation could generate a broader isolated area after PVI with a cryoballoon.^4^, ^8^, ^9^, ^10^ Complete conduction block at the LA roof line was reportedly achieved in 81.0%–95.0% of ablations with RF energy^11^, ^12^ and in 88.0%–99.8% with a cryoballoon.^4^, ^8^ Regarding the chronic status of the lesions, the reported durability of the LA roof line was 37.5%–72.0% with RF energy^13^, ^14^ and 74.5% with a cryoballoon.^6^ Indeed, several reports have shown that additional LAPW ablation results in better clinical outcomes,^8^, ^15^ but the adjunctive value of LAPW ablation in addition to PVI is controversial.^16^ In our study, the LA roof line was successfully ablated with a cryoballoon in 95.7% of the patients, of whom 72.5% had confirmed durable lesions, which is consistent with previous reports. Moreover, further analysis regarding the recurrence type in this study revealed that LAPW ablation, in addition to PVI, could reduce the persistent type of AF recurrence, which appeared to be clinically important. A prior examination investigated the atrial substrate size by utilizing the parameter of conduction velocity, refractory period, and LA body area.^17^ Considering the mechanism of reduced persistence of AF after ablation based on the reported theory, cryoballoon ablation of the LA roof might be effective by reducing the atrial substrate size because it could produce a broader scar area, as previously reported.^4^, ^6^, ^9^ In this regard, cryoballoon ablation might be preferable to a conventional RF method for creating the LA roof line. AF persistence appeared to depend on the site of AF drivers, and it might be an important factor where AF drivers exist; however, findings regarding AF drivers were not examined in this study. Moreover, in this study, the value of A wave velocity measured by transthoracic echocardiography was significantly lower in patients who underwent cryoballoon ablation of the LAPW, although those who underwent cryoballoon ablation of the LAPW appeared to have compatible A wave velocity according to a previous study.^18^ Further studies might be required to assess atrial function after the ablation more precisely and select candidates who should undergo the cryoballoon ablation of the LAPW.

One of the reasons for hesitation in performing linear ablation was the possibility of iatrogenic AT occurrence after the ablation procedure. After PVI, including both RF energy and cryoballoon ablation, AT recurrence was observed in approximately 10% of the patients, according to previous reports.^19^, ^20^, ^21^ In a study that compared the prevalence of AT recurrence between patients who underwent PVI with RF energy and those who underwent PVI with a cryoballoon, the AT recurrence rate tended to be lower in those who underwent PVI with a cryoballoon.^19^ Among recurrent ATs, perimitral AT was reportedly the most frequently observed, accounting for approximately 30% of AT recurrences in previous investigations.^20^, ^21^ Regarding linear ablation with RF energy, the reported recurrence rate of AT originating from the LA was 4% in patients.^3^ Data regarding the prevalence of AT recurrence after linear ablation with a cryoballoon are limited. Aryana et al.^22^ investigated the recurrence type after cryoballoon ablation of the LAPW. They demonstrated that 65.2% of the patients who received repeat ablation procedures (15.6% of the patients who had undergone cryoballoon ablation of the LAPW in the index ablation procedure) experienced AT recurrence. In our study, 8.6% (18/210) of the patients who underwent cryoballoon ablation of the LAPW had AT recurrence, which is comparable with previous reports, including RF ablation. We demonstrated that perimitral AT was the most frequent type of recurrent AT, accounting for half of the identified recurrent ATs in our study cohort. LA roof‐dependent AT was confirmed in only three patients, whom all underwent cryoballoon ablation for LAPW in the index ablation. However, considering previous reports in which the prevalence of LA roof‐dependent AT recurrence was 1%–2% after PVI with a cryoballoon,^20^, ^21^ the recurrence rate of the LA roof‐dependent AT in this study might not be high. The high durability of the lesions created at the LAPW might contribute to the low prevalence of LA roof‐dependent AT recurrence.

Our study had a few limitations. First, this was a single‐center, non‐randomized retrospective study. Therefore, the results regarding additional cryoballoon ablation of the LAPW should be carefully interpreted, although no significant differences were found in patient characteristics such as LAD or the existence of left common PV or right middle PV between patients who underwent cryoballoon ablation of the LAPW and those who did not. Finally, the use of AADs after the index ablation was also different among study patients; thus, clinical follow‐up data require prudent interpretations. A randomized multicenter study with a fixed protocol is necessary to confirm the efficacy and influence of LAPW ablation with a cryoballoon. Nonetheless, we presented detailed data in terms of acute and long‐term success rates and clinical outcomes after ablation, including the recurrence type. We consider that these findings will contribute to further examinations regarding cryoballoon ablation of the LAPW in the future.

## 5 CONCLUSIONS

In this study, the efficacy of cryoballoon ablation for the LAPW, especially at the LA roof, was demonstrated. A sufficient success rate of complete conduction block at the LA roof, including the chronic durability of the lesions, can be expected. AT recurrence after ablation does not increase in patients who undergo cryoballoon ablation of the LAPW. The recurrence rate of the persistent type of AF after ablation becomes lower when cryoballoon ablation of the LAPW is performed.

The conversion from the persistent to the paroxysmal type after ablation may have various benefits. The risk of stroke or heart failure might be reduced, and complete elimination of AF might be possible after an additional trigger‐based ablation. Therefore, the strategy of LAPW ablation could lead to better clinical outcomes after ablation in patients with non‐paroxysmal AF who would not benefit from PVI alone. However, identifying suitable candidates for additional LAPW ablation remains to be clarified.

## CONFLICTS OF INTEREST

Authors declare no conflict of interests for this article.

## ETHICS DISCLOSURE

The protocol for this research project has been approved by a suitably constituted Ethics Committee of the institution and it conforms to the provisions of the Declaration of Helsinki. Committee of Japan Red Cross Yokohama City Bay Hospital, Approval No. 2018‐81. All informed consent was obtained from the individuals.
